# Frequency of Human Papilloma Virus (HPV) subtypes 31,33,35,39 and 45 among Yemeni women with cervical cancer

**DOI:** 10.1186/s13027-015-0026-9

**Published:** 2015-09-07

**Authors:** Hussain Gadelkarim Ahmed, Saleh Hussein Bensumaidea, Ibraheem M. Ashankyty

**Affiliations:** Molecular Diagnostics and Personalized Therapeutics Unit, University of Ha’il, Ha’il, Kingdom of Saudi Arabia; Department of Pathology, College of Medicine, University of Ha’il, 2240 Hai’l, Kingdom of Saudi Arabia; Department of Histopathology and Cytology, FMLS, University of Khartoum, Khartoum, Sudan; Department of Pathology, College of Medicine, Hadhramout University of Science and Technology, Hadhramout, Yemen; Department of Clinical Laboratory Sciences, College of Applied Medical Sciences, University of Ha’il, Ha’il, Kingdom of Saudi Arabia

**Keywords:** Cervical cancer, HPV31, HPV33, HPV35, HPV39, HPV45, Yemeni

## Abstract

**Background:**

Identification of different HPV subtypes in unidentified communities provides sufficient information for screening and monitoring potential impact of a vaccination program. Therefore, the aim of this study was to screen for the presence of HPVs subtypes 31,33,35,39 and 45 among Yemeni women with Cervical Cancer.

**Methodology:**

A total of 200 (150 malignant and 50 benign) tissue samples were obtained from Yemeni women with cervical cancer, were investigated for the presence of HPV subtypes 31,33,35,39 and 45 by Polymerase Chain Reaction (PCR).

**Results:**

Of the 150 cervical cancer tissue specimens, HPV 31, HPV 33, HPV35, HPV 39 and HPV45 were identified in 10/150 (6.7 %), 6/150 (4 %), 6/150 (4 %), 5/150 (3.3 %) and 10/150 (6.7 %), respectively. The frequency of these HPV subtypes among Yemeni women with cervical cancer was 24 %. Conclusion: HPV 31, HPV 33, HPV35, HPV 39 and HPV45 were prevalent among Yemeni women with cervical cancer.

## Introduction

Cervical cancer is the third most common cancer in women Worldwide [1]. Oncogenic human papillomavirus (HPV) is the most important risk factor associated with cervical cancer [2]. HPV have been divided into high- and low-risk on the basis of their oncogenic potential. High risk HPV is considered to be the leading etiological cause for cervical cancer [3]. Cervical cancer is one of the most common cancers among women worldwide [4]. Cervical cancer is a very slow progressive disease which needs prolonged period to progress from normal to pre-cancer and to the potentially fatal invasive cancer. There is a gap of 10–20 years between pre-cancer and cancer which offers an opportunity to screen, detect and treat pre-cancer and avoid its progression to cancer. Immunocompromised women, however, progress more frequently and more quickly to pre-cancer and cancer [5, 6].

Infection with high-risk human papillomavirus (HR-HPV) is established as the main cause of cervical carcinoma [7]. HPV has been involved in 99.7 % of cervical squamous cell cancer cases worldwide [8]. Human Papilloma Virus (HPV) is small DNA virus commonly infecting mucosa and cutaneous keratinocytes. Up to now, at least 200 HPV genotypes are known. HPV have been divided into high- and low-risk on the basis of their oncogenic potential. High risk HPV is considered to be the leading etiological cause for cervical cancer [3]. Low-risk HPV types include types 6, 11, 42, 43, and 44. High-risk HPV types include types 16, 18, 31, 33, 35, 39, 45, 51, 52, 56, 58, 59, 68, 73, and 82 [9].

The L1 and L2 proteins form icosahedral capsids for progeny virion generation. Based on sequence variation of the L1, L2, HPV variants differ in biological and chemical properties, as well as geographical distribution [10–12]. Intratypic sequence variation has also been found in the E2, E4, E5, E6, and E7 genes of HPV-16 [10]. The E6 and E7genes encode viral oncoproteins that target Rb and p53, respectively. During the viral life cycle, these proteins facilitate stable maintenance of episomes and stimulate differentiated cells to reenter the S phase [12]. The oncogenicity of specific HPV variants appears to vary geographically ethnically [10, 11]. In 2012, the International Agency for Research on Cancer concluded that there was consistent and sufficient epidemiological, experimental and mechanistic evidence of carcinogenicity to humans for HPV31, HPV33, HPV35, HPV39, HPV45, for cervical cancer. Consequently, these types were regarded as 1A carcinogens. They all belong to the family of the α-Papillomaviridae, in specific to the species, α7 (HPV39, HPV45) and α9 (HPV31, HPV33, HPV35) [13].

However, there are very limited studies about HPVs and their related cancers in the majority of developing countries such as the greater Middle East which including Yemen and its neighboring countries, such as Saudi Arabia, Qatar, United Arab Emirates [14]. However, most of the studies in this context have focused on HPV subtypes 16 and 18 ignoring other high risk or low risk HPV subtypes. Therefore, the aim of this study was to find out the prevalence of HPV 31, HPV 33, HPV35, HPV 39 and HPV45 among Yemeni women with cervical cancer.

## Materials and methods

In this study, 200 formalin fixed paraffin wax processed tissue samples of cervical lesions were obtained from formerly operated patients from National Cancer Centers and different histopathology laboratories in Sana’a, Hadhramout and Aden, Yemen. All specimens and data were obtained from the archive of National Cancer Centers, and different hospitals and different private histopathology laboratories in the three governorates mention above during the period from 2008 to 2012 (full coverage). Cancer specimens included: Squamous Cell Carcinoma (SCC), Adenocarcinoma (Ad), Adenosquamo-Carcinoma (AdSC).

### DNA extraction

DNA was extracted from paraffin-embedded samples, by immersing tissue section in xylene to dissolve the paraffin from the tissue, and then rehydrated using a series of ethanol washes. Proteins and harmful enzymes such as nucleases were digested by proteinase K. Buffer containing denaturing agent (sodium dodecyl sulfate (SDS)), was added to facilitate digestion [15]. Nucleic acids were purified from the tissue lysate using buffer-saturated phenol and high speed centrifugation. Following phenol extractions, RNase A was added to eliminate contaminating RNA. Additional phenol extractions following incubation with RNase A were used to remove any remaining enzyme. Sodium acetate and isopropanol were added to precipitate DNA, and high speed centrifugation was used to pellet the DNA and facilitate isopropanol removal. Washing with 70 % ethanol was performed to remove excess salts, followed by centrifugation to re-pellet the DNA [16, 17]. DNA is re-suspended in distilled water, quantified and stored at −20 °C Purified DNA was subsequently used in downstream applications of PCR.

### DNA quantification

To evaluate the DNA quantification after DNA extraction, we had analyzed DNA measurement using a NanoDrop spectrophotometer.

### Amplification of HPV

Type specific primers (primer for HPV 31, HPV 33, HPV35, HPV 39 and HPV45) were used to detect HPV31, 33, 35, 39 and 45 DNA in cervical benign and malignant lesions, adopting the classification procedure of the American Joint Committee on Cancer (AJCC) TNM classification and the International Federation of Gynecology and Obstetrics (FIGO) staging system for cervical cancer [18]. Amplification was performed according to HPV31, 33, 35, 39 and 45 kit from Sacace-Biotechnologies S.r.l. Caserta –Italy. The final reaction volume of 40 μl containing 20 μl mix-1 (contained in PCR tubes), 10 μl of mix-2 and 10 μl of extracted DNA (sample). Negative control, and positive HPV31,33,35,39 and 45 DNA tubes contained 10 μl of DNA buffer, 10 μl of HPV31,33,35,39 and 45 were applied.

Samples and controls were amplified using Gene Amp PCR system 9700. The PCR programis described in Table 1.

**Table 1:** PCR steps. Cycle's time temp PCR steps

Step	Temperature °C	Time	Cycles
1	95 °C	Pause	
2	95 °C	15 min	1
3	95 °C	30 s	42
	63 °C	30 s	
	72 °C	40 s	
4	72 °C	1 min	1
5	10 °C	Storage	

### Gel-electrophoresis

The PCR products were visualized in 2 % Agarose gel with 0.5 μg/ml Ethidium bromide. Ten micro liters of 100 bp DNA ladder and PCR product was loaded on the gel. Gel electrophoresis was performed at 120 V and 36 mA for 60 min. Pictures were taken by Gel documentation system (Gel mega, digital camera and software in a computer).

### Interpretation of PCR results

According to manufacture HPV31, 33, 35, 39 and 45 kit (Sacace-Biotechnologies S.r.l. Caserta –Italy) manual, the PCR product length for HPV 31 should be 520 bp, HPV 33 should be 227 bp, HPV 35 should be 280 bp, HPV 39 should be 340 bp, HPV 45 should be 475 bp.

### Data analysis

Data management was done by using the Statistical Package for Social Sciences (SPSS version 16). SPSS was used for analysis and to perform Fisher exacttest for statistical significance (P value <0.05 was considered significant). The 95 % confidence level and confidence intervals were used.

### Ethical consideration

Before the study conducted the proposal of the study were ethically approved by ethical committee of the Sudan University of Science and Technology. Then the inform consent were agreed by the general managers of all national cancer centers, hospitals and private histopathology laboratories in which the study were performed.

## Results

**Fig. 1:**
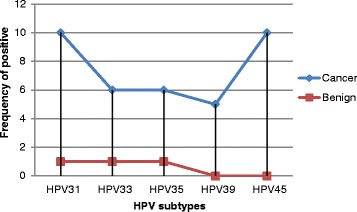
Description of HPV subtypes by Lesion type

In this study we investigated 200 formalin fixed paraffin wax tissue blocks obtained from patients previously presented with cervical lesions (150 were diagnosed with cervical cancer and 50 with benign cervical lesions). The ages of the studied group were ranging from 21 to 75 years with a mean age of 47 years. Of the 150 cervical cancer tissue specimens, HPV 31, HPV 33, HPV35, HPV 39 and HPV45were identified in 10/150 (6.7 %), 6/150 (4 %), 6/150 (4 %), 5/150 (3.3 %) and 10/150 (6.7 %), respectively. Of the 50 benign cervical lesions tissue specimens, HPV 31, HPV 33, HPV35, HPV 39 and HPV45 were identified in 1/50 (2 %), 1/50 (2 %), 1/50 (2 %), 0/50 (0 %) and 1/50 (2 %), respectively, as indicated in Fig. 1.

Co-infection was only identified between HPV31 and HPV45, in two cases (one malignant and one benign), which gives a prevalence of 36/150 (24 %) for HPV subtypes (31,33,35 and 45).

**Fig. 2:**
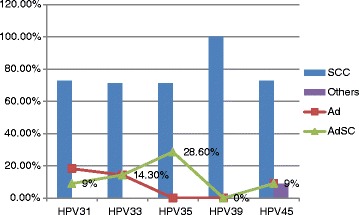
Description of cervical cancer type by HPV subtype

Table 2 Summarizesthe distribution of the cervical cancer cases by type of cancer and HPV subtype. Most of HPV subtypes were identified within squamous cell carcinoma tissues. HPV31, HPV33, HPV35, HPV39 and HPV45, were identified in 8/11 (72.7 %), 5/7 (71.4 %), 5/7 (71.4 %), 5/5 (100 %), 8/11 (72.7 %) respectively, as indicated in Fig. 2.

**Table 2:** Distribution of the cervical cancer cases by type of cancer and HPV subtype

Cancer	HPV31		HPV33		HPV35		HPV39		HPV45	
	+ve	-ve	+ve	-ve	+ve	-ve	+ve	-ve	+ve	-ve
SCC	8	116	5	119	5	119	5	119	8	116
Ad	2	17	1	18	0	19	0	19	1	18
AdSC	1	2	1	2	2	1	0	3	1	2
Others	0	4	0	3	0	4	0	4	1	3
Total	11	139	7	143	7	143	5	145	11	139

The frequencies of infection rates among different age groups were summarized in Table 3. For HPV31, the highest infection rate was found among age groups 36–45 years followed by 46–55 and 56+, representing 6/11 (54.4 %), 4/11 (36.4 %) and 1/11 (9 %), respectively. For HPV33, the highest infection rates were seen among age groups <35 years, followed by 36–45, and 46–55 years, representing 3/7 (42.9 %), 2/7 (28.6 %) and 2/7 (28.6 %). Regarding HPV35, the highest infection rates were seen among age ranges, 56+ years representing3/7 (42.9 %) followed by 45–55, 36–45 and <35 years, constituting 2/7 (28.6 %), 1/7 (14.3 %) and 1/7 (14.3 %), respectively. HPV39, has showed high rates of infection among age ranges <35 and 46–55 years representing 2/5 (40 %) for each. HPV45 has denoted high infection rates among age groups 36–45 years, representing 4/11 (36.4 %), followed by age ranges 56+,<30 and 46–55 years, constituting 3/11 (27.3 %), 2/11 (18.2 %) and 2/11 (18.2 %), in this order.

**Table 3:** Distribution of HPV subtypes by age

Age	HPV31		HPV33		HPV35		HPV39		HPV45	
	+ve	-ve	+ve	-ve	+ve	-ve	+ve	-ve	+ve	-ve
>35	0	31	3	28	1	3	2	29	2	29
36-45	6	54	2	57	1		0	60	4	56
46-55	4	61	2	70	2	59	2	70	2	70
56+	1	36	0	37	3	69	1	36	3	34
Total	11	189	7	193	7	193	5	195	11	189

**Fig. 3:**
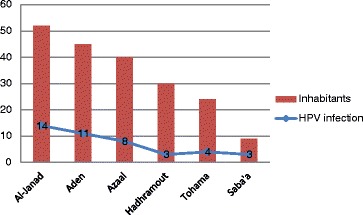
Description of the study population by HPV infection and residence

**Fig. 4:**
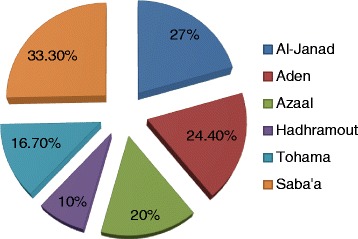
Description of the study population by HPV infection within entire residence group

With respect to the residence, most of cases of Cervical Cancer were coming from Al-Janad representing 52/200 (26 %) followed by Aden constituting 45/200 (22.5 %), as shown in Fig. 3. In regard to the residence and HPV infection, the great majority of infections were identified among Al-Janad populations, representing 14/43 (32.6 %) followed by Aden, Azaal, Tohama Hadhramout and Saba’a, constituting 11/43 (25.6 %), 8/43 (18.6 %), 4/43 (9.3 %), 3/43 (7 %) and 3/43 (7 %), respectively, as shown in Table 4, Fig. 3. However, when calculating the percentage within individual entire residence, the highest proportion of infection was found in the Saba’a representing 33.3 % followed by Al-Janad, Aden, Azaal, Tohama and Hadhramout constituting 27, 24.4, 20, 16.7 and 10 %, in this order, as indicated in Fig. 4.

**Table 4:** Distribution of the HPV subtypes by residence

Region	HPV31		HPV33		HPV35		HPV39		HPV45	
	+ve	-ve	+ve	-ve	+ve	-ve	+ve	-ve	+ve	-ve
Azaal	2	36	1	39	2	38	1	39	4	36
Aden	3	42	0	45	2	44	2	43	4	41
Hadhramout	1	29	1	29	0	30	0	30	1	19
Al-janad	4	48	2	50	3	50	2	50	3	49
Tohama	1	24	3	21	0	24	0	24	0	24
Saba’a	1	8	1	8	1	8	0	9	0	9
Total	12	188	8	192	8	192	5	195	12	188

## Discussion

The mortality of cervical cancers is high in Yemen and most cases are diagnosed late stage of the disease. In fact, there is a complete paucity of data in this context from Yemen; due to the absence of sufficient national registries. Although, the prevalence of HPV is unknown in Yemen, but data from the neighboring countries showed increased burden. In present study, the relationship between cervical cancer and the presence of the high risk HPV (HR-HPV) subtypes HPV31, HPV33, HPV35, HPV39, and HPV45 genome was tested. Data on HPV type spreading is essential to assess the possible impact of present and future HPV preventive strategies.

The prevalence and distribution of HPV genotypes differs significantly globally, and these variances might be related to the complex geographical and biological interaction between different HPV types and host immunogenetic factors.

However, there is a lack of literature regarding the burden of HPV in Yemen. To the best of our knowledge there was only two study investigated the prevalence of HPV in cervical cancer. The first one examined the presence of HPV16 and HPV18 in 84 cervical cancer tissue samples that were previously categorized as harboring HPV by immunohistochemistry using K1H8 (anti-HPV). In the use of PCR testing for HPV subtypes 16 and 18; 62/84 (73.8 %) were identified positive for HPV subtype 16 and the remaining 22/84 (26.2 %) were negative. Then again, on testing the specimens for HPV subtype 18; 23/84 (27.4 %) were found positive and the remaining 61/84 (72.6 %) were revealed negative [19]. Another study from Yemen found that the prevalence of HPV subtypes 52,56,58,59, and 66, among cases was 0.6, 0, 4, 3.3 and 0 % respectively [20].

Again the presence of the high risk HPV (HR-HPV) subtypes HPV31, HPV33, HPV35, HPV39, and HPV45 was previously reported in a number of studies. Prospective follow-up studies have proposed that persistent HR-HPV infections play a crucial role in the progression of CIN lesions and in the development of cervical cancer. The mean clearance time for the distinct HR-HPV type was 16.5 months. HPV16 and HPV31 were the most persistent infections (clearance times = 18.1 and 16.2 months, respectively), whereas HPV39 infections cleared quickly. The mean copies per cell in HPV18/45, HPV31, HPV33/52/58, and HPV39 infections were higher in persisting HPV infections than in HPV infections that cleared, but the difference was not significant [21]. In a study from Kuwait, investigated the distribution of HPV in 298 women with abnormal cervical cytology, found that the prevalence of HPV33 was 9.9 % [22], which was very high compared to our findings. Although we found that the most prevalent HPV subtypes were HPV31 and HPV45, a recent study has shown that, the most prevalent HPV genotype, HPV31 followed by HPV35 [23].

Regarding the relationship between age and HPV infection in this study, it was observed that HPV31 and HPV45 were relatively seen in younger ages compared to HPV35 and HPV39; hence, HPV33 was more frequent among elder women. However, the number of infected women was so little to generate specific age range for the studied women.

Moreover, there were considerable discrepancies regarding the distribution of the different HPV subtypes among different regions. However, the explanations of these findings require further onsite assessment of different behavioral, social and other demographical measures. A comprehensive screening for HPV subtypes linked to Pap. Test may render more sufficient results for future planning of potential vaccination program in Yemen.

However, the limitation of the present study include its investigation included limited HPV subtypes.

In conclusion: HR-HPV31, HR-HPV33, HR-HPV35, HR-HPV39, and HR-HPV45 are prevalent among Yemeni women with cervical cancer. Further, measures and strategic plans are urgently required to reduce the burden of cervical cancer in Yemen.
